# Case of Naso-Pharyngeal Polypi—Ecrasement and Osteo-Plastic Operation—Rapid Recovery

**Published:** 1880-04

**Authors:** 


*Aberdeen Royal Infirmary.—Case of Naso-Pharyngeal Polypi; Ecrasement and Osteo-Plastic Operation; Rapid Recovery. (Under the care of Dr. J. C. Ogilvie Will.)*

J. B——, aged seventeen, a fisherman, was admitted on May 4th, 1878, suffering from nasal polypus. The patient stated that about two years and a half ago he fell among rocks, receiving a severe blow upon his nose which caused great hæmorrhage, and was followed by considerable swelling. Six months afterwards he began to experience difficulty in breathing, and suffered from other symptoms of nasal occlusion. The symptoms increasing, and frequent attacks of bleeding supervening, he consulted a medical man, who injected astringent solutions, and extracted a small polypus. Sometime afterwards he was advised to seek admission to the infirmary, and when he did so the following symptoms were noted—namely, nose much deformed, flattened, and broadened, presenting the appearance of frog-face; right nostril completely blocked up by a tumor which was visible through the anterior nares; the soft palate was depressed and much displaced forward by a hard, firm swelling; deglutition difficult; speech indistinct; breathing during sleep labored and stertorous; sense of smell impaired.

Attempts to determine the points of attachment of the polypi were attended with great difficulty, on account of the lad's nervousness, and after the endeavor which was made to obtain the required information, he made preparations for returning home, and was only prevented from fulfilling his intention by the promise that he should be subjected to no further examination, so that the only information which it was possible to obtain was that the tumor seen through the anterior nares was attached to the floor of the nasal fossa, and the probability of the polypus being a branched one, consisting of two portions springing from a single stem, one passing forward and the other backward, seemed to both Dr. Will and his colleagues to be the most probable state of the parts.

On May 7th the wire rope of an écraseur was passed through the anterior nares into the pharynx, and appeared in the throat, *not* between the tumor and the posterior wall of the pharynx, as it was concluded that it would do from the view that had been taken of the point of attachment of the polypus, but in front of the tumor, or between it and the velum, which indicated that there was opposition to the passage of the wire loop behind, and that the pharyngeal portion of the morbid growth sprang from the base of the skull, and not from the floor of the nasal fossa. This was verified by the next step of the procedure, which consisted in passing the loop attached to the écraseur over the tumor, and then making slight traction on the handle of the instrument, when it was evident that the neck of the tumor was encircled by the wire. The wire was then gradually tightened in the ordinary manner, and in the course of a few moments a polypus the size of a large walnut was removed from the mouth, and the écraseur was then withdrawn. It was now evident that the polypus, which had projected behind and had been removed, was perfectly distinct and separate from the tumour seen through the anterior nares, for it was still *in situ*. Dr. Will therefore now passed his forefinger into the nostril, and found that it was filled with a large hard tumor, having the attachment to the floor, which he had previously ascertained. He made an endeavor to enucleate it by means of his finger-nail, but the area of attachment was so broad, and the adhesions were so firm, that his efforts were fruitless.

A consultation with Drs. Ogston and Garden was then held, when it was determined to cut through and turn aside the right superior maxillary bone along with the overlying soft parts, in order to gain access to the point of origin of the growth. The bone and soft parts were accordingly detached after the manner of Langenbeck, and free access being thus afforded to the point of attachment of the mass, it was readily extirpated by avulsion and the free use of curved scissors. The bone was then replaced and the soft parts were united by sutures. Hæmorrhage, which was excessive during the operation, was restrained by the application of catgut ligatures to the vessels as they were cut, and by the insertion of a plug of lint into the nostril; no dressings were applied. The polypus thus removed was irregular in shape, somewhat flattened, and about the size of a small tomato. Professor Stirling examined it, and reported that its structure was that of an ordinary œdematous fibrous tumour, with retentive cysts due to obstructed mucous glands. At 4 p. m. the patient was sensible, but exhausted ; pulse extremely feeble. No chloroform sickness. Ordered a teaspoonful of brandy every two hours, and ice to suck. 8 p. m.: Had slept a little; did not complain of pain. Pulse stronger, 132; temperature 100.3°. To have an injection of morphia if required.

May 10th.—9 a. m.: Had passed a quiet night without hypodermic injection. No complaint of pain, but stated to nurse that he was already much relieved by the operation, as breathing was so much easier, although the plugs of lint were still in the nostril. Pulse 116 ; temperature 100°.

11th.—Morning: Passed a somewhat restless night. Parts swollen, and right eye completely closed from œdema. Pulse 113; temperature 101°. Evening: Swelling much lessened.

Union of cut parts complete; all the sutures removed. Pulse 108; temperature 101.1°.

From this date everything went well, the swelling steadily disappearing, the pulse improving in strength and decreasing in frequency, the temperature, which never reached a higher point than 101.2°, speedily becoming normal, and the patient's general condition undergoing so rapid an improvement that on May 18th he was allowed to leave bed, and on the 23rd he was able to go out of doors. For some weeks there was a free discharge of pus from the nostril, which was frequently washed out with an antiseptic solution. One small point in the upper line of incision gave way, and remained open for a considerable period after his return home, but, on the extrusion of a small plate of bone, it speedily healed. On June 8th the lad had left hospital in the enjoyment of perfect health, the wound, with the exception of the small point above mentioned, being soundly healed, while the maxillary bone was firm and immovable.

Six months afterwards, when he presented himself at the hospital, he stated that he could breathe with equal ease through either nostril, and careful examination of the parts previously affected by disease failed to reveal the presence of any morbid appearances, while his face, although disfigured by linear cicatrices, was a not unpleasant looking one, and bore but comparatively slight evidences of the very severe procedure which had been put in force.

